# Hyperoside induces ferroptosis in chronic myeloid leukemia cells by targeting NRF2

**DOI:** 10.1186/s10020-024-01002-7

**Published:** 2024-11-21

**Authors:** Junyi Wei, Quanyou Chai, Yuqiao Qin, Long Li, Chunling Guo, Zhaoyang Lu, Huimin Liu

**Affiliations:** 1 Institute of Immunology, Zhejiang University School of Medicine, 866 Yuhangtang Road, Hangzhou, 310058 China; 2 Liangzhu Laboratory, Zhejiang University Medical Center, 1369 West Wenyi Road, Hangzhou, China; 3 Department of Hematology, The Second Hospital of Shanxi Medical University, Taiyuan, China; 4 Department of Cardiology, Sir Run-Run Shaw Hospital, Zhejiang University School of Medicine, Hangzhou, China; 5 Key Laboratory of Cardiovascular Intervention and Regenerative Medicine of Zhejiang Province, and Engineering Research Center for Cardiovascular Innovative Devices of Zhejiang Province, Hangzhou, China; 6 Department of Cardiology, The Second Hospital of Shanxi Medical University, Taiyuan, China

**Keywords:** Hyperoside, Chronic myeloid leukemia, Ferroptosis, NRF2

## Abstract

**Background:**

Hyperoside (quercetin-3-O-β-D-galactopyranoside) is a flavonol glycoside compound derived from plants in the Hypericum and Crataegus genera that reportedly exhibits an array of anti-inflammatory, antioxidant, and antitumor properties such that it has been used to treat various diseases. Whether it can serve as an effective treatment for chronic myeloid leukemia (CML) cells, however, has yet to be established. The present study was thus devised to assess the therapeutic effects of hyperoside on CML cells and to clarify the underlying mechanism of action.

**Methods:**

Cellular viability, proliferative activity, migration, and apoptotic death were respectively analyzed through CCK-8, EDU, transwell, and flow cytometry assays. RNA-seq and bioinformatics approaches were further employed to evaluate the mechanisms through which hyperoside influences CML cells, while analyses of reactive oxygen species (ROS) and free iron were detected with commercial kits. Transmission electron microscopy was used to assess mitochondrial morphology. Molecular docking, cellular thermal shift assay (CETSA), and drug affinity responsive target stability (DARTS) approaches were also used to explore the ability of hyperoside to target NRF2.

**Results:**

From a mechanistic perspective, hyperoside was able to inhibit SLC7A11/GPX4 signaling in a manner that was abrogated by the ferroptosis inhibitor ferrostatin-1. NRF2 was also closely associated with the inactivation of the SLC7A11/GPX4 axis mediated by hyperoside such that overexpressing NRF2 ablated the benefits associated with hyperoside treatment.

**Conclusions:**

The present analyses indicate that hyperoside can target the NRF2/SLC7A11/GPX4 axis to induce ferroptotic CML cell death.

**Supplementary Information:**

The online version contains supplementary material available at 10.1186/s10020-024-01002-7.

## Introduction

Chronic myeloid leukemia (CML) is a highly proliferative form of cancer derived from hematopoietic stem cells harboring the Ph chromosome and BCR::ABL fusion gene positivity as a result of the fusion of the ABL and BCR genes on chromosomes 9 and 22, respectively. This BCR::ABL fusion oncoprotein serves as a key driver of CML development although its ability to activate a range of mitotic signaling pathways (Skorski et al. 1997). Following the advent of tyrosine kinase inhibitors (TKIs), CML patients experienced clinical improvements such that they are now a cornerstone of the clinical management of this tumor type (Hochhaus et al. 2020). However, imatinib and second-generation TKI resistance is a persistent challenge in many patients that limits long-term benefits (Boulos et al. 2011). TKI therapy also fails to benefit many patients with CML owing to drug reactions or intolerance (Druker et al. 2006), underscoring the need to develop alternative treatment for the treatment of CML derived from natural products or synthetic agents.

Hyperoside (quercetin-3-galactoside) is a flavonol glycoside that is primarily produced by plants in the Hypericum genus (Middleton et al. 2000; Zou et al. 2004). It has been demonstrated to exert antitumor properties when treating a range of human tumor cell types including breast (Sun et al. 2020), lung (Zhou et al. 2021), prostate (Chen et al. 2024), bladder (Yang et al. 2024), cervical (Guo et al. 2019), ovarian (Zhu et al. 2017, 2022) and renal cancer cells (Zhou et al. 2021). Several mechanisms through which hyperoside can induce tumor cell death have been documented, including the reactive oxygen species(ROS)-mediated activation of NF-κB signaling and consequent apoptotic death (Qiu et al. 2019), as well as the suppression of the oncogenic microRNA-27a to impair proliferative activity (Li et al. 2014). Little research to date, however, has focused on how hyperoside affects leukemia cells, and no data are thus available to clarify the degree to which hyperoside can induce the ferroptotic death of CML cells or the underlying molecular mechanisms responsible for such activity.

The cysteine/glutamate transporter System x_c_^−^ exists as a heterodimer composed of heavy chain (4F2, encoded by SLC3A2) and a light chain (xCT, encoded by SLC7A11) components (Pope and Dixon 2023). The overexpression of SLC7A11 is commonly observed in many types of cancer (Koppula et al. 2018), and SLC7A11 has been established as a key inhibitor of ferroptotic signaling (Yan et al. 2023). The key SLC7A11 downstream target glutathione peroxidase 4 (GPX4) can utilize glutathione to facilitate a reduction in the hydroperoxidation of lipids, ultimately leading to the inhibition of ferroptosis (Xue et al. 2023). A growing body of evidence indicates that SLC7A11/GPX4 signaling activity can promote the progression of cancer by limiting ferroptotic induction (Chen et al. 2021). The NRF2 transcription factor is a key driver of SLC7A11 transcriptional activity (Rojo et al. 2018). While the NRF2/SLC7A11/GPX4 signaling axis is closely tied to tumorigenesis (Wen et al. 2023), its relevance to the anti-leukemia effects of hyperoside has yet to be documented.

This study was designed to evaluate the ability of hyperoside to protect against CML in vitro, together with complementary analyses of the underlying mechanisms responsible for this protective activity. These analyses ultimately revealed that hyperoside was able to induce the ferroptotic death of CML cells through the targeting of the NRF2/SLC7A11/GPX4 pathway. This is thus the first report documenting a novel mechanism underlying the anti-CML activity of hyperoside, emphasizing its potential utility as a candidate treatment for this form of cancer.

## Materials and methods

### Cell culture

K562 and Meg-01 CML cells (ATCC, VA, USA) were cultured in RPMI-1640 (Gibco, NY, USA) with 10% fetal bovine serum (FBS, Gibco) and 1% penicillin/streptomycin in a 37℃ thermostat incubator (ThermoFisher, USA) under 5% CO_2_. Cells were assessed for growth every other day, and media was routinely changed. STR validation and mycoplasma testing were performed before experimental use.

### Reagents

Z-VAD-FMK (HY-16658B), E-necrosulfonamide (HY-100573), 3-Methyladenine (HY-19312), Deferoxamine mesylate (HY-B0988), Ferrostatin-1 (HY-100579) and imatinib (HY-15463R) were from MedChemExpess. Hyperoside (C_21_H_20_O_12_, MW: 464.38, HPLC ≥ 98%) was from Nanjing Spring & Autumn Biological Engineering (Nanjing, China).

### CCK-8 assay

To analyze cell viability with a CCK-8 kit (Dojindo, Kumamo, Japan), they were plated in 96-well plates (5,000/well) and treated with hyperoside at different concentrations (0, 0.5, 1, 2 mM) or by addition of different concentrations of inhibitors Z-VAD-FMK (20 µM), 3-Methyladenine (5 mM), necrosulfonamide (1 µM), or ferrostatin-1 (2µM) for 24 h. To determine whether hyperoside could enhance the effect of imatinib, the cells were treated with imatinib (0, 1, 2 µM) or hyperoside (0, 0.5, 1, 2 mM) for 24 h. Then, each well was supplemented with 10 µL of CCK-8 solution, followed by a further 2–4 h incubation at 37℃. Absorbance at 450 nm was then analyzed with an appropriate instrument.

### EdU-594 staining assay

An EdU-594 staining kit (Beyotime, Shanghai, China) was used to measure the proliferation of CML cells. After incubating Meg-01 and K562 cells for 24 h with various hyperoside concentrations (0, 0.5, 1, 2 mM) in 6-well plates, they were fixed for 30 min with 4% paraformaldehyde (PFA), permeabilized for 5 min with 0.3% Triton X-100 in PBS, and stained for 2 h with 1× EdU working solution. After counterstaining for 5 min using DAPI at room temperature, fluorescence microscopy was used to detect EdU-positive cells, which were further analyzed in ImageJ.

### Transwell migration assay

A 24-well transwell chamber (Corning, MA, USA) with an 8 μm porous membrane filter was used to evaluate CML cell migration. Following treatment for 24 h with hyperoside (0, 0.5, 1, 2 mM), 1 × 10^4^ of these cells were suspended in serum-free media and added to the upper portion of each transwell insert, while the lower chamber was filled with complete media containing 10% FBS. Following incubation for a further 24 h, cells in the upper chamber were discarded, while 4% PFA was used to fix cells that had migrated across the membrane for 20 min, followed by a 20 min staining step with crystal violet, and the use of a cotton swab to remove any cells on the upper membrane surface. Random fields of view were then imaged under a microscope, and ImageJ was used for image analysis.

### Apoptosis analyses

After treating CML cells for 24 h with hyperoside (0, 0.5, 1, or 2 mM), they were rinsed twice in PBS, and an Annexin V-FITC/PI apoptosis detection kit (BD Biosciences, CA, USA) was used as directed. Briefly, after suspending cells in 100 µL of 1×binding buffer, they were stained with 5 µL each of Annexin V-FITC and PI for 15 min while protected from light at room temperature. After adding 400 µL of 1×binding buffer, apoptotic cell frequencies were quantified on an FC 500 flow cytometer (Beckman, USA).

### Western immunoblotting

RIPA buffer was used to extract total protein from CML cells, with a BCA assay then being used to quantify protein levels before separating samples via SDS-PAGE and transferring them onto PVDF membranes. A room temperature blocking stem with 5% non-fat milk (1 h) was followed by incubation at 4℃ with primary antibodies specific for β-actin, GPX4, SLC7A11, BAX, and BCL02 (all diluted 1:1000; Proteintech, Wuhan, China) overnight. Membranes were probed for 1 h with HRP-linked secondary antibodies, after which ECL signal detection was performed with a ChemiDoc XRS + Imaging System (Bio-Rad), and densitometric quantification was conducted with ImageJ.

### RNA sequencing

Trizol (ThermoFisher Scientific, MA, USA) was used to extract total RNA, while cDNA library sequencing was performed with the Illumina HiSeq X Ten platform. The criteria of Q value < 0.05 and fold change > 2 or fold change < 0.5 were used for the identification of differentially expressed genes (DEGs).Differential expression analyses were implemented with the R DESeq (2012) package, and OE Biotech (Shanghai, China) performed all data analyses. The RNA-seq data have been deposited in GEO with the accession number GSE280885.

### GSH, MDA, and NADPH analyses

Intracellular GSH and GSSG concentrations, MDA levels, and NADPH levels were detected with appropriate commercial kits (Beyotime) used as directed.

### qPCR

After using Trizol to extract CML cell RNA, cDNA was prepared and qPCR was performed with a PrimeScriptRT Reagent Kit (Takara). Relative expression was quantified using the 2^−△△Ct^ method, and GAPDH was used for normalization. Sangon Biotech (Shanghai, China) provided all primers (Supplementary Table S1).

### Intracellular ROS, lipid peroxidation, Fe^2+^, and MitoSOX analyses

Following a 24 h treatment with hyperoside (2 mM), cells were stained for 30 min using 10 µM DCFH-DA (Beyotime), 2 µM C11-BODIPY (ThermoFisher Scientific), 1 µM Ferroorange working solution (Dojindo, Kumamo, Japan), or 5 µM MitoSOX (Yeasen, Shanghai, China), followed by DAPI counterstaining and imaging with a fluorescence microscope.

### Transfection and transduction

Lentiviral vectors for the overexpression of NRF2 and corresponding negative controls (NC) were purchased from GenePharma (Shanghai, China). Cells were transduced with these viruses at an appropriate multiplicity of infection (MOI) based on the cell and viral titers, followed by a 12–16 h incubation at 37 °C. Cells from each well were then centrifuged (2 min, 2,000 rpm) and the supernatant was exchanged for fresh culture medium. At 72 h post-infection, green fluorescent protein (GFP) expression was assessed as a measure of transduction efficiency. Cells were incubated in the presence of puromycin (5 µg/mL) in complete medium which was exchanged every 3–4 days to select for infected cells. This process was sustained until no puromycin-induced killing was observed and the transfection efficiency reached 100%. All transduction steps were performed as directed by the manufacturer.

### Transmission electron microscopy

After fixing cells with 2.5% glutaraldehyde for 1 h, they were centrifuged and treated as directed, followed by analysis with a transmission electron microscope.

### Molecular docking

Binding between hyperoside and NRF2 was assessed via a molecular docking analysis in which NRF2 (ID: AF-Q16236-F1) served as the receptor and was downloaded from the alphafold database (https://alphafold.com/). Hyperoside structural information was downloaded from PubChem (https://pubchem.ncbi.nlm.nih.gov/) and served as the ligand. The online AutoDock Vina-based CB-Dock2 tool (https://cadd.labshare.cn/cb-dock2/php/index.php) was used to conduct docking analyses, while PyMOL 2.6 and PLIP (Protein-Ligand Interaction Profiler) were employed for visualization.

### Drug affinity responsive target stability (DARTS) assay

After collecting cells, 250 µL of NP-40 was added on ice for 30 min, followed by centrifugation (15,000 rpm, 15 min, 4℃). Supernatants were then quantified via BCA assay, and lysates were separated into five portions treated using DMSO or various hyperoside concentrations (0, 0.5, 1, or 2 mM) for 2 h at room temperature, followed by incubation for 30 min in the presence of pronase (2.5 µg/mL) at 37℃, Then, 5× loading buffer was added at an appropriate volume (0.25× protein volume), samples were boiled for 10 min at 100℃, and NRF2 expression was assessed via Western immunoblotting.

### Cellular thermal shift assay (CETSA)

After suspending 1 × 10^7^ K562 cells in PBS with 1% protease and phosphatase inhibitors, they were snap-frozen for 30 s in liquid nitrogen, thawed at room temperature, and this cycle was repeated three times, after which samples were centrifuged in a fresh tube (15,000 rpm, 20 min, 4℃), and supernatants were collected. These samples were then equally divided into two parts which were treated for 30 min with DMSO or hyperoside (2 mM) at 25℃, followed by the separation of these samples into 8 aliquots each that were respectively heated for 3 min at 46, 49, 52, 55, 58, 61, 64, and 67℃. Following a room temperature incubation for 3 min, these tubes were placed on ice, centrifuged (15,000 rpm, 30 min, 4 °C), and supernatants were collected and added to an appropriate amount of 5× loading buffer, followed by boiling in a water bath for 5 min to facilitate protein denaturation.

### Statistical analysis

Data are means ± SD from experiments performed at least three times. GraphPad Prism 8 was used for all analyses, with results having been compared with Student’s t-tests and one-way ANOVAs. *P* < 0.05 was regarded as significant.

## Results

### Hyperoside induces the death of CML cells and suppresses their viability and migration

**Fig. 1:**
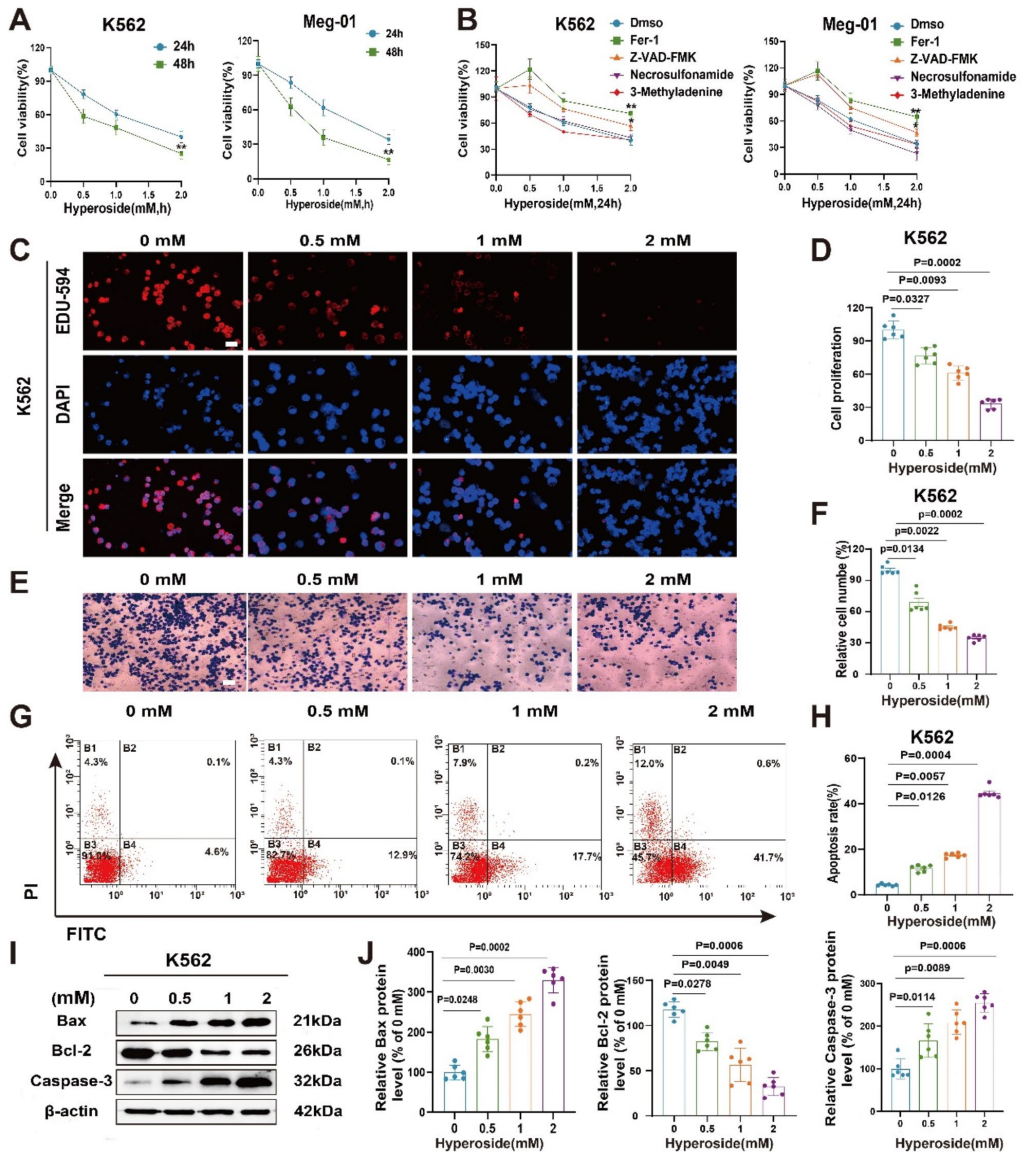
Hyperoside impairs CML cell viability, migration, and proliferation. **A** Following hyperoside treatment for various time periods, K562 and Meg-01 cell viability was quantified. **B** The viability of K562 and Meg-01 cells following hyperoside treatment (0, 0.5, 1, 2 mM) for 24 h with or without Z-VAD-FMK (20 µM), 3-Methyladenine (5 mM), necrosulfonamide (1 µM), or ferrostatin-1 (2µM) was analyzed. **C-D.** Proliferation was detected through an EdU-594 assay. Scale bar: 50 μm. **E-F.** Transwell assays were used to measure migration. Scale bar: 100 μm. **G-H.** Flow cytometry was used for the quantitative analysis of apoptosis. **I-J.** Bax, Bcl-2, and Caspase-3 protein levels were detected via Western immunoblotting

A CCK-8 assay demonstrated that hyperoside was able to suppress CML cell viability in a dose-dependent fashion (Fig. 1A), with respective IC50 values of 1.435 mM and 1.343 mM in the K562 and Meg-01 cell lines. To determine the mode of cell death induced by hyperoside, cells were treated with necrosulfonamide, ferrostatin-1 (Fer-1), Z-VAD-FMK, and 3-MA to respectively inhibit necroptosis, ferroptosis, apoptosis, and autophagy. Fer-1 treatment restored the viability of hyperoside-challenged CML cells (Fig. 1B), whereas Z-VAD-FMK only partially improved the viability of these cells. The ability of hyperoside to enhance the sensitivity of CML cells to imatinib was also investigated. CML cells were treated with different concentrations of imatinib and hyperoside for 24 h. The results showed that the combination of hyperoside and imatinib could significantly reduced the viability of CML cells (Fig. S1a-b).

Consistently, in EdU-594 assays, hyperoside was found to significantly impair K562 and Meg-01 cell proliferation (Fig. 1C-D, Fig. S1c-d), while also suppressing the migration of these cells in a transwell assay (Fig. 1E-F, Fig. S1e-f).

Flow cytometry also demonstrated that hyperoside readily induced higher levels of apoptosis than control treatment (Fig. 1G-H, Fig. S1g-h), while Western immunoblotting indicated that hyperoside increased proapoptotic protein levels (Caspase-3, Bax) and lowered antiapoptotic Bcl-2 levels (Fig. 1I-J, Fig. S1 i-j). Together, these data indicate that hyperoside can serve as a significant inhibitor of CML cell growth and migration, inducing the death of these cells.

### Hyperoside induces ferroptotic CML cell death

**Fig. 2:**
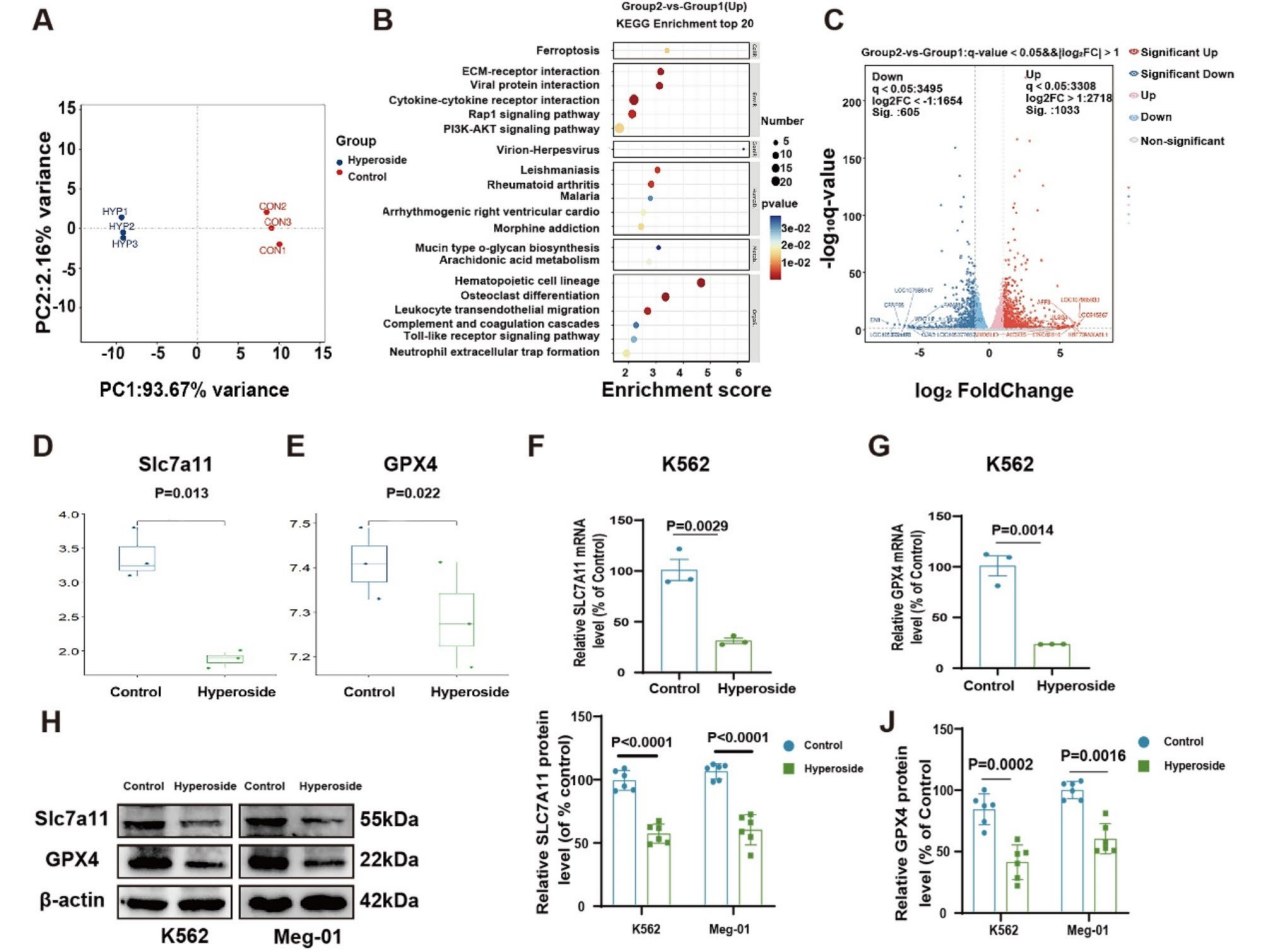
Hyperoside can trigger ferroptotic activity within CML cells. **A** Sequencing data were used to conduct PCA analyses. **B** KEGG enrichment analyses of genes differentially expressed in K562 cells following hyperoside treatment. **C** A volcano plot of differentially expressed genes. **D-E.** Sequencing analyses of SLC7A11 and GPX4 mRNA levels. **F-G.** SLC7A11 and GPX4 mRNA levels as measured via qPCR in K562 cells. **H-J.** SLC7A11 and GPX4 protein levels were detected via Western immunoblotting in K562 and Meg-01 cells

To gain further insight into how hyperoside shapes CML cell development, transcriptomic sequencing was conducted in the control group and hyperoside (2 mM) groups. PCA analyses revealed the clear separation of sample groups (Fig. 2A), and KEGG enrichment analyses indicated that genes upregulated in hyperoside-treated cells were enriched in the ferroptosis pathway (*P* < 0.05)(Fig. 2B). Volcano plots revealed that hyperoside treatment was associated with the respective downregulation and upregulation of 605 and 1,033 genes relative to the control group (|log2FC| > 1, q < 0.05) (Fig. 2C). The SLC7A11/GPX4 pathway has been firmly established as a key mediator of ferroptosis. Both SLC7A11 and GPX4 were found to be downregulated in the hyperoside group (Fig. 2D-E), and hyperoside was confirmed to significantly lower SLC7A11 and GPX4 mRNA (Fig. 2F-G) and protein (Fig. 2H-J) levels within treated CML cells. These data highlight the ability of hyperoside to serve as a driver of ferroptotic CML cell death through its ability to inhibit SLC7A11/GPX 4 pathway signaling.

### Hyperoside induces ROS biogenesis, lipid peroxidation, and mitochondrial damage in CML cells

**Fig. 3:**
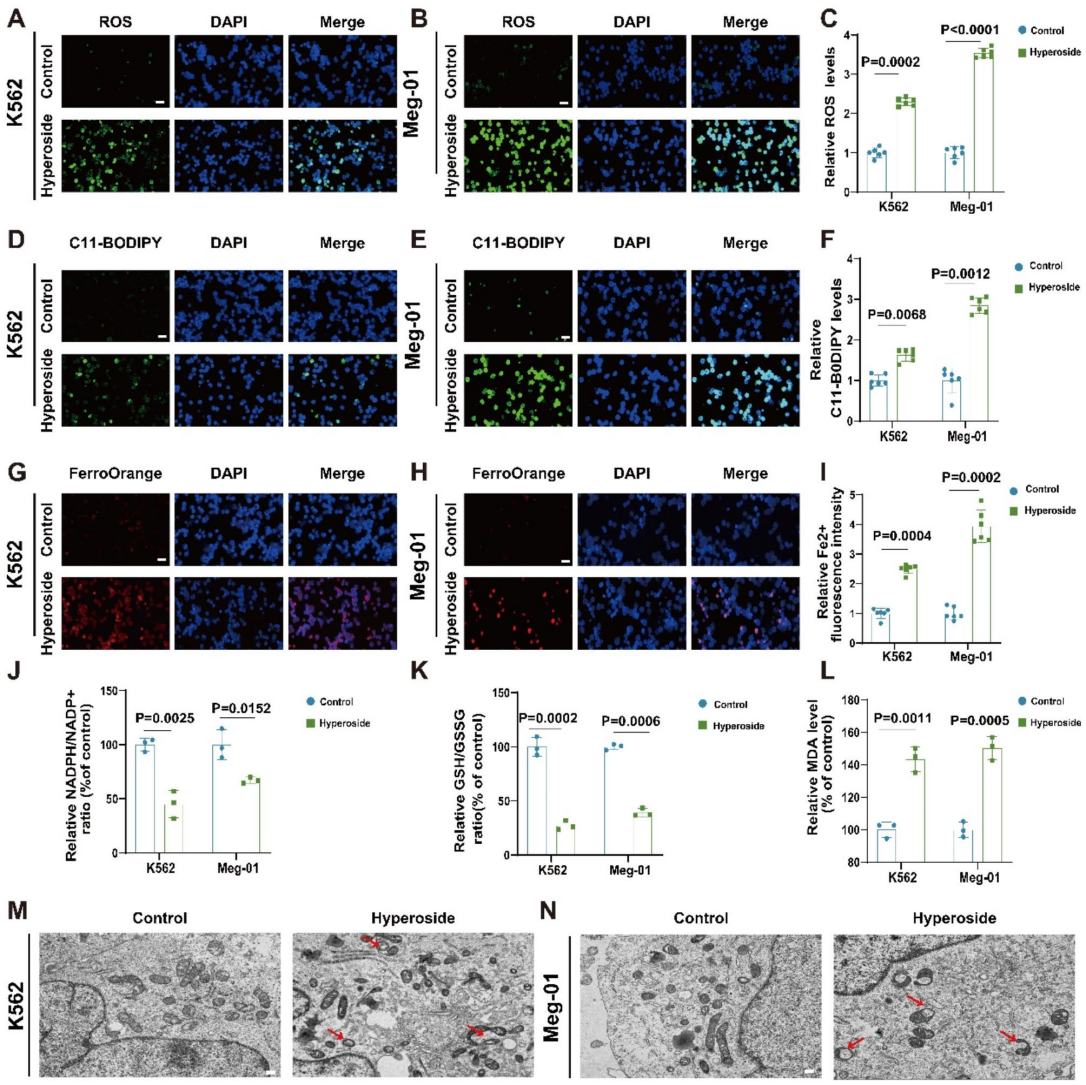
Hyperoside enhances lipid peroxidation, ROS production, and mitochondrial damage within CML cells. **A-C.** Intracellular ROS within K562 and Meg-01 cells were detected via DCFH-DA staining. Scale bar: 50 μm **D-F.** C11-BODIPY 581/591 staining was used to detect lipid ROS levels in K562 and Meg-01 cells. Scale bar: 50 μm. **G-I.** FerroOrange staining was used to detect labile iron in K562 and Meg-01 cells. Scale bar: 50 μm. **J-L.** The NADPH/NADP + ratio **J**, GSH/GSSG ratio **K**, and MDA levels **L** in K562 and Meg-01 cells were assessed with appropriate assay kits. **M-N.** Treated K562 and Meg-01 cell mitochondria were analyzed via TEM. Red arrows indicate mitochondrial damage induced by ferroptosis.Scale bar: 2 μm

ROS is produced as a by-product of oxidative metabolism and represents a general term for peroxide-containing oxygen free radicals and other free radicals. Marked increases in ROS levels can cause significant damage to cellular structures. ROS levels in cells were measured using a ROS detection kit. It was found that the intensity of green fluorescence was significantly increased in the hyperoside group, indicating that hyperoside increased ROS levels in CML cells, resulting in cellular damage(Fig. 3A-C).

Ferroptosis is a recently discovered form of cell death characterized by the accumulation of Fe2 + and damage to mitochondrial membranes caused by oxidized polyunsaturated fatty acids (Tang et al. 2021). To further investigate the effect of hyperoside on ferroptosis, the levels of lipid peroxidation in CML cells were measured using the lipid peroxidation probe C11-BODIPY, indicating significant increases in the hyperoside group compared with the control group(Fig.  3D-F). Iron plays a central role in the pathogenesis of ferroptosis (Toyokuni et al. 2017). and the FerroOrange Fe^2+^ probe was thus used to detect intracellular unstable iron. Hyperoside treatment was found to increase the FerroOrange fluorescence signal in both CML cell lines, consistent with Fe2 + accumulation (Fig. 3G-I). Ferroptosis is also characterized by glutathione (GSH) depletion (Kim et al. 2023). and corresponding analyses of GSH and NADPH levels revealed that both the GSH/GSSG and NADPH/NADP + ratios were reduced in CML cells in response to treatment using hyperoside (Fig. 3J-K). Hyperoside also induced the accumulation of the lipid peroxidation byproduct MDA (Fig. 3L).

The level of mitochondrial ROS is an important index for evaluating the degree of mitochondrial damage (Li et al. 2023).The Mitosox probe was used to determine the production of mitochondrial ROS. Similarly, MitoSOX staining showed that treatment with hyperoside significantly increased mitochondrial ROS levels (Fig. S2a-d). We also examined the effect of hyperoside on ferroptosis-associated morphological characteristics. The unique morphological features associated with ferroptosis include a reduction in mitochondrial volume and an increase in membrane density (Stockwell et al. 2020). Transmission electron microscopy (TEM) revealed significant ferroptosis-related morphological changes in K562 and Meg-01 cells after treatment with hyperoside, visible as thickening, discontinuity, and shrinkage of the mitochondrial membranes (Fig. 3M-N). These results suggest that hyperoside triggers ferroptosis in CML cells.

### Ferroptosis is necessary for the anti-leukemic activity of hyperoside in CML

**Fig. 4:**
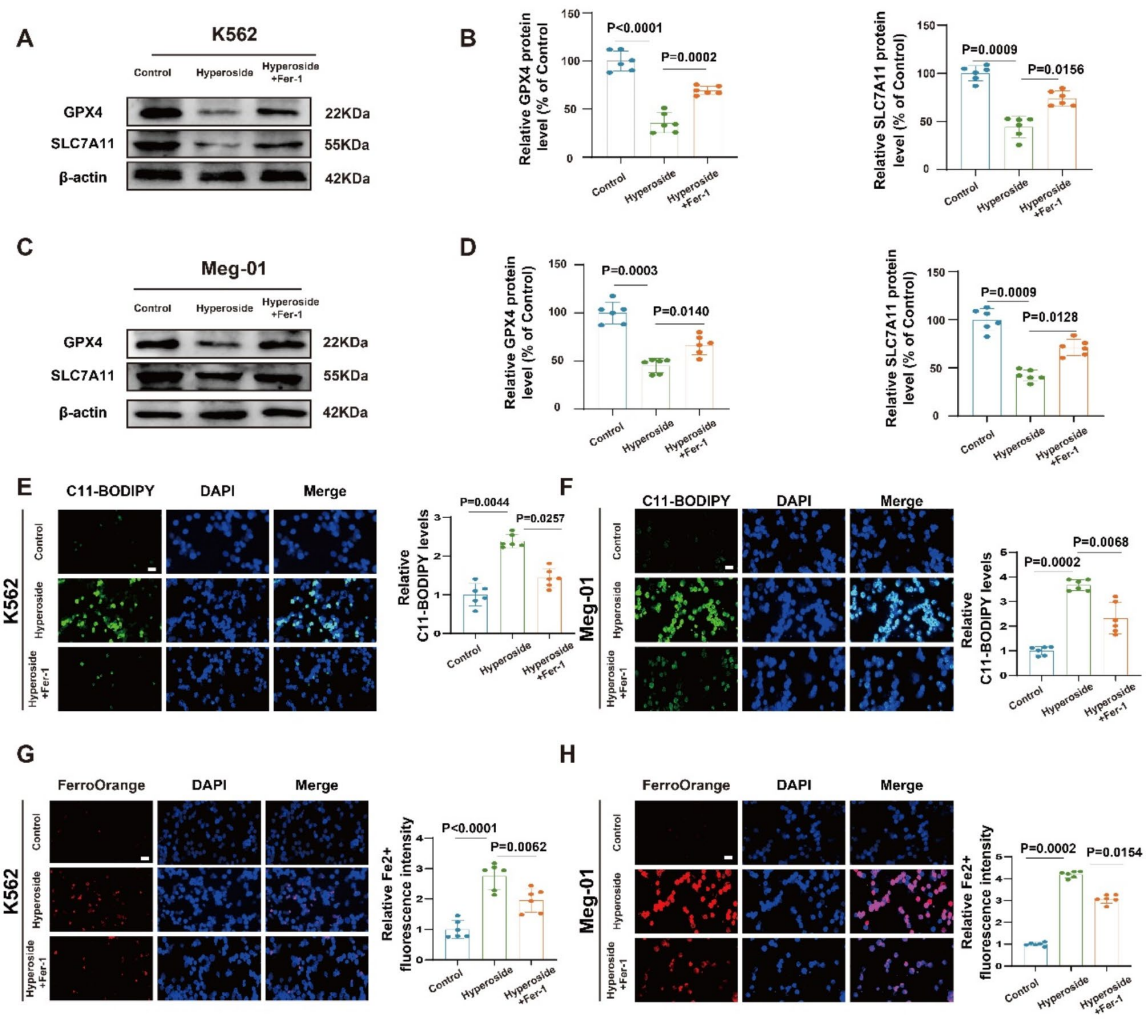
Ferroptosis inhibitors abrogate the antitumor effects of hyperoside treatment in CML cells. **A-D.** SLC7A11 and GPX4 protein levels were detected via Western immunoblotting within K562 and Meg-01 cells. **E-F.** Lipid ROS detection within K562 and Meg-01 cells was performed via C11-BODIPY 581/591 staining. Scale bar: 50 μm. **G-H.** Labile iron was detected in K562 and Meg-01 cells via FerroOrange staining. Scale bar: 50 μm

To more fully explore the role of ferroptosis in the antitumor activity of hyperoside, ferroptosis inhibitors were next used in an effort to rescue these antitumor effects. Western immunoblotting revealed that Fer-1 abrogated the inactivation of the SLC7A11/GPX4 pathway by hyperoside (Fig. 4A-D). Fer-1 was also able to reverse the accumulation of lipid ROS and Fe^2+^ within CML cells treated using hyperoside (Fig. 4E-H), while additionally suppressing MDA production and reversing the hyperoside-associated changes in the GSH/GSSG and NADPH/NADP + ratios (Fig.S3a-f). Ferroptosis is thus required for the anti-leukemic activity of hyperoside.

### Hyperoside can induce ferroptosis through the inhibition of NRF2/SLC7A11/GPX4 signaling within CML cells

**Fig. 5:**
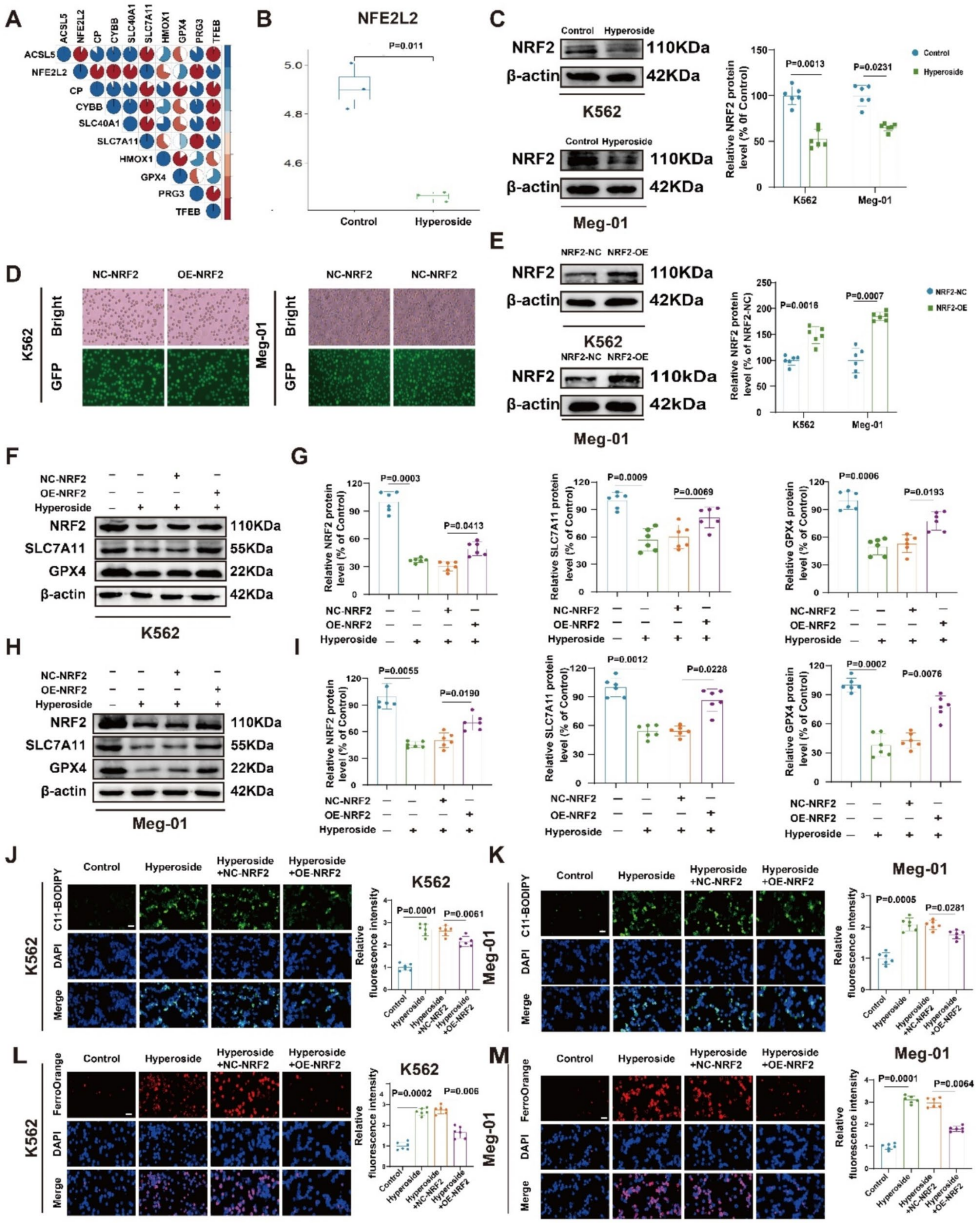
Hyperoside suppresses NRF2/SLC7A11/GPX4 signaling within CML cells. **A** Sequencing results revealed a positive correlation between SLC7A11 and NRF2. **B** NRF2 mRNA levels. **C.** NRF2 was detected in K562 and Meg-01 cells via Western immunoblotting. **D-E.** NRF2-overexpressing cell line construction. **F-I.** NRF2, SLC7A11, and GPX4 in K562 and Meg-01 cells were detected via Western immunoblotting. **J-K.** Lipid ROS detection within K562 and Meg-01 cells was performed via C11-BODIPY 581/591 staining. Scale bar: 50 μm. **L-M.** Labile iron was detected in K562 and Meg-01 cells via FerroOrange staining. Scale bar: 50 μm

NRF2 serves as a key regulator of SLC7A11/GPX4 signaling, which suppresses ferroptosis (Dodson et al. 2019). Sequencing analyses revealed a positive correlation between SLC7A11 and NRF2 (Fig. 5A). Hyperoside is capable of inhibiting NRF2 mRNA expression (Fig. 5B), and decreases in NRF2 protein levels following hyperoside treatment were confirmed in both cell lines via Western immunoblotting (Fig. 5C). GFP fluorescence imaging was used to verify the transduction efficiency in cells after purinomycin selection (Fig. 5D).

Lentiviruses were used to prepare NRF2 overexpression cell lines (Fig. 5E), and the ability of upregulation to reverse hyperoside-induced NRF2, SLC7A11, and GPX4 downregulation was thus demonstrated (Fig. 5F-I). Overexpressing NRF2 also led to the elimination of hyperoside-induced intracellular lipid peroxide and labile iron accumulation (Fig. 5J-M). These data demonstrated the central role of the NRF2/SLC7A11/GPX4 pathway in hyperoside-induced ferroptosis in CML.

### Hyperoside binds to NRF2 to induce ferroptosis in CML cells

**Fig. 6:**
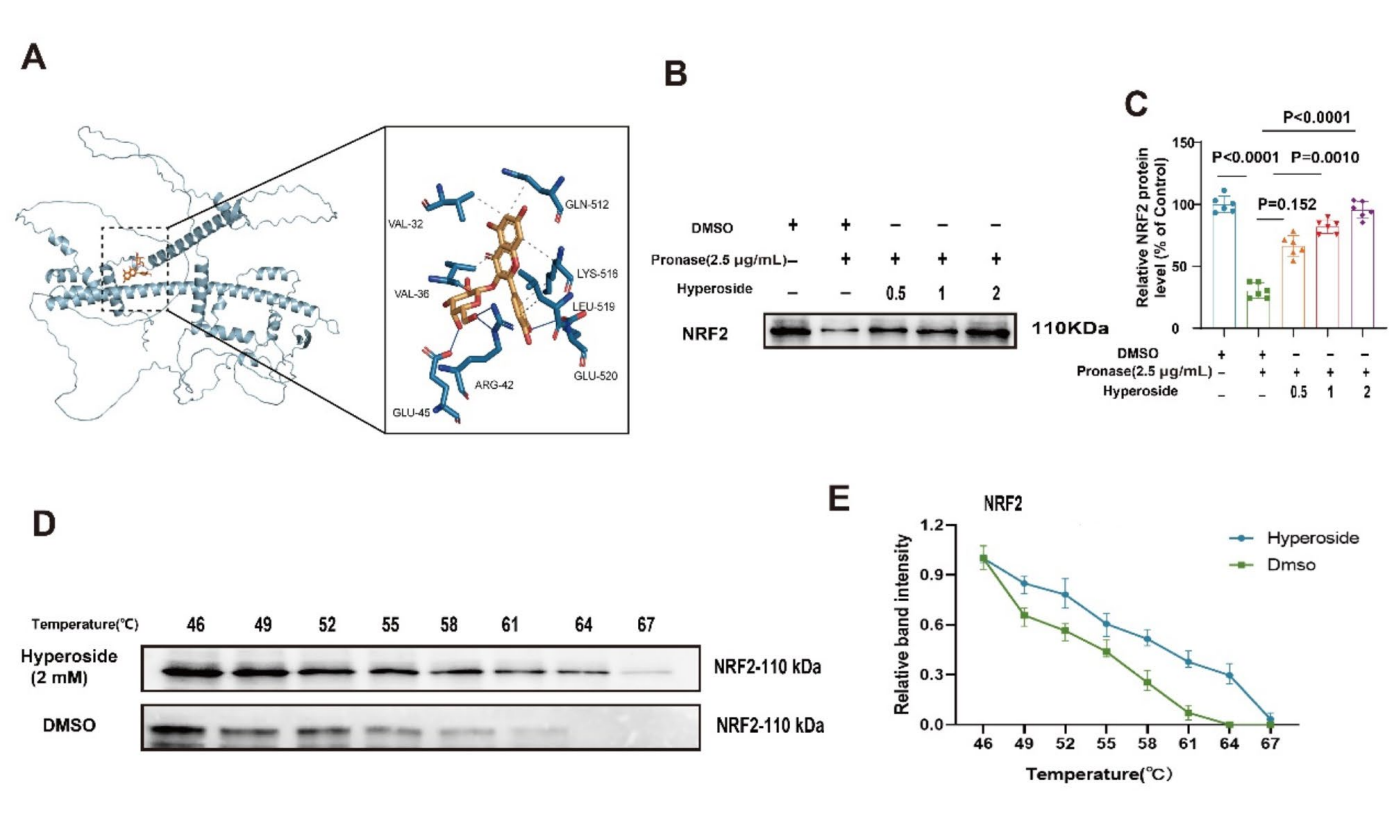
Hyperoside and NRF2 physically interact. **A.** Molecular docking analyses of interactions between hyperoside and NRF2. **B-C.** NRF2 protein levels in DARTS analyses. **D-E.** NRF2 protein levels in CETSA analyses

Lastly, a series of analyses were conducted to determine whether hyperoside is capable of interacting with the NRF2 protein within CML cells. Molecular docking analyses were thus conducted, revealing a binding energy of -7.7 kcal/mol for the interaction between hyperoside and NRF2 consistent with stable binding (Fig. 6A). DARTS analyses also indicated that hyperoside binding can protect NRF2 from protease-mediated digestion, slowing the degradation of this transcription factor (Fig. 6B-C). CETSA showed that hyperoside stably bound to NRF2 and attenuated its degradation rate at high temperature.(Fig.6D-E) (Fig. 6D-E).

## Discussion

**Fig. 7:**
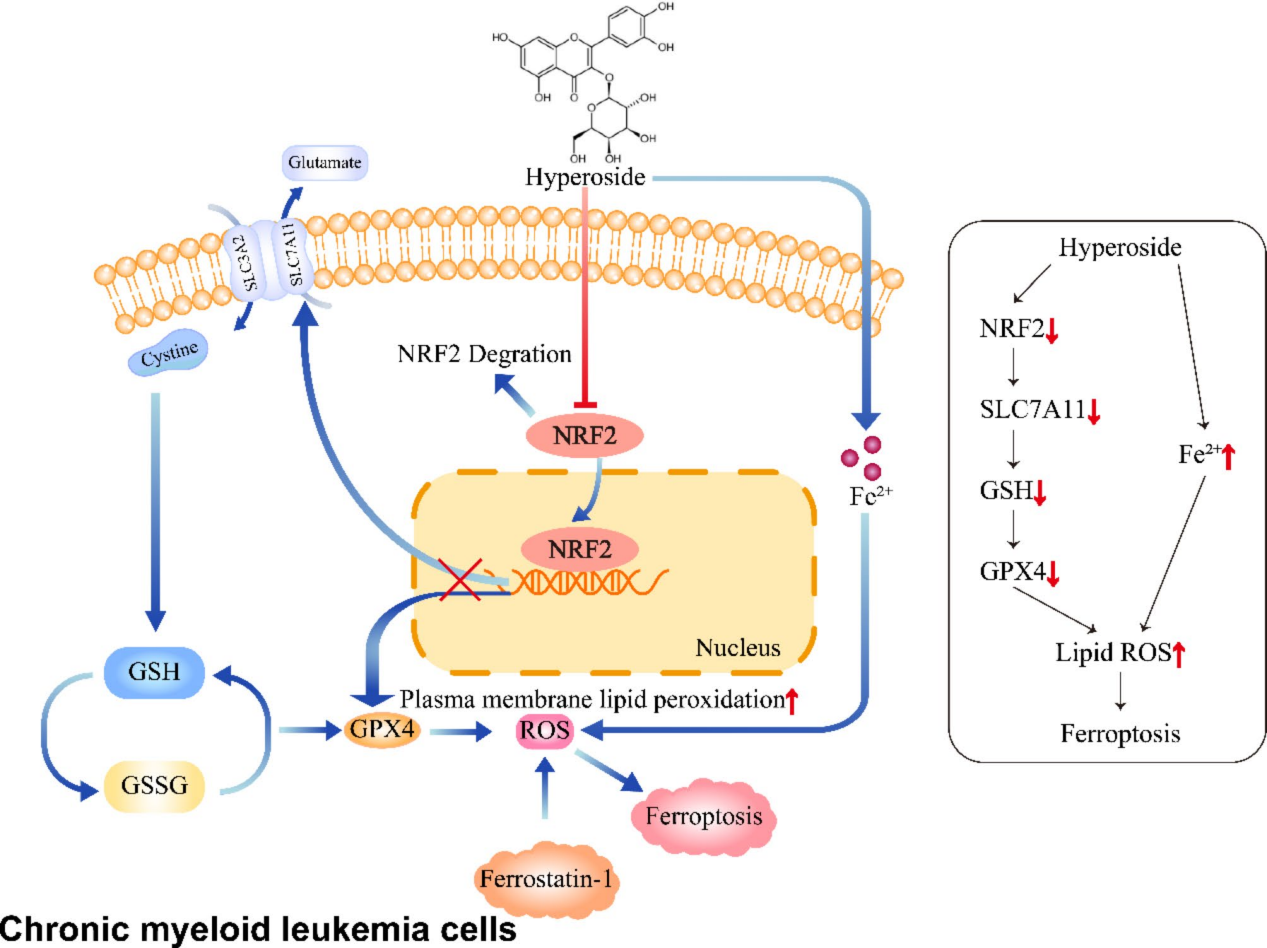
Schematic overview of the main study results. Hyperoside can induce CML cell ferroptosis through targeting of the NRF2/SLC7A11/GPX4 pathway

CML is a malignant myeloproliferative disease that accounts for an estimated 14% of all cases of leukemia newly diagnosed among adults (Jabbour et al. 2023; Siegel et al. 2019). The BCR::ABL TKI imatinib is a first-line treatment for CML, but some patients exhibit imatinib resistance (Vetrie et al. 2020). There is thus a clear need to identify alternative therapeutic options. Interest in extracting bioactive ingredients from herbal medicines has grown in recent years (Shanmugam et al. 2017). Hyperoside exhibits a range of antitumor activities through its ability to suppress angiogenesis, induce apoptosis, inhibit proliferation, and initiate cell cycle arrest in many cancer cell lines (Wang et al. 2022). It has been reported that hyperoside inhibits the AKT/mTOR/P70S6K pathway while activating the ERK1/2 pathway to induce autophagy in A549 lung cancer cells (Fu et al. 2016). Hyperoside also promotes apoptosis in thyroid squamous cell carcinoma cell lines through the Fas/FasL signaling pathway (Liu et al. 2017) In this study, the mechanisms through which hyperoside affects CML cells were thus analyzed. Hyperoside was ultimately found to exert its anti-leukemic effects through the induction of NRF2/SLC7A11/GPX4 pathway-mediated ferroptosis within CML cells (Fig. 7).

Ferroptosis and apoptosis are both types of programmed cell death that are closely intertwined, with apoptosis potentially giving rise to ferroptotic death in some instances, while ferroptosis can also render cells more sensitive to apoptotic death (Tong et al. 2022). The ferroptotic and apoptotic pathways are thus promising targets for the treatment of cancer. Many drugs capable of treating cancer have been reported to function through the dual targeting of these pathways (Zhang et al. 2022). For instance, curcumin can induce ferroptosis and apoptosis in osteosarcoma cells through its ability to modulate the NRF2/GPX4 pathway (Yuan et al. 2023). Dihydroisotanshinone I can similarly induce these forms of cell death in breast cancer cells (Lin et al. 2019), while oridonin can do so in osteosarcoma cells to suppress their growth (Zhang et al. 2024). In this study, hyperoside was found to induce the ferroptotic and apoptotic death of CML cells, suggesting it exerts its antitumor effects through these two interrelated processes. Distinct from necrosis, autophagy, or apoptosis, ferroptosis is defined by iron ion and lipid peroxide accumulation (Yan et al. 2021). In recent years, ferroptosis has been widely leveraged to guide the treatment of many cancer types, and it has been established as a process that generally suppresses tumor growth (Liang et al. 2019). GPX4 can regulate ferroptosis, protecting against this form of cell death through the glutathione-mediated elimination of phospholipid peroxides (Forcina and Dixon 2019). The absence of inhibition of GPX4 can spur ferroptosis induction together with a rise in lipid ROS within cells, inhibiting tumor cell proliferative activity (Jia et al. 2020). Many flavonoids have been reported to have antioxidant properties, which may account for their antitumor and apoptosis-inducing effects (Khan et al. 2012). Tumor cells with higher ROS levels were more susceptible to the induction of cell death than normal cells with lower ROS levels. Therefore, novel anticancer drugs that promote ROS production may have significant potential. Hyperoside induces G2/M cell cycle arrest and apoptosis in human colorectal cancer cells by increasing ROS accumulation (Zhang et al. 2017). Hyperoside has been shown to increase intracellular ROS levels in a dose-dependent manner in A549 cells (Chen et al. 2020). This is consistent with our findings. Inhibitors of GPX4 including RSL3 and FIN56 can induce its degradation within tumor cells, leading to ferroptotic initiation (Imai et al. 2017; Wei et al. 2020). SLC7A11 is an important multichannel transmembrane protein with cystine/glutamate reverse transporter protein activity involved in the Xc system (Koppula et al. 2018). Here, hyperoside was found to suppress SLC7A11 and GPX4 expression within CML cells while also altering the levels of ROS, Fe^2+^, MDA, NADPH, and GSH in these cells in a manner that could be reversed by inhibitors of ferroptosis. Ferroptosis thus appears to play an important role in hyperoside-mediated anti-CML activity.

NRF2 is a transcription factor capable of regulating an array of antioxidant enzymes and genes to preserve redox homeostasis (Basak et al. 2017), and it is commonly upregulated in human cancers. Both GPX4 and SLC7A11 are transcriptional targets of NRF2 (Sasaki et al. 2002). RNA-seq and Western immunoblotting analyses confirmed the ability of hyperoside to inhibit NRF2 expression, and positive correlations were observed between NRF2 and SLC7A11. NRF2 overexpression, however, impaired the ability of hyperoside to inhibit SLC7A11 and GPX4. Molecular docking, DARTS, and CETSA experiments also demonstrated the high degree of affinity of hyperoside for NRF2. These data thus support a model wherein hyperoside can control CML cell ferroptosis through the inhibition of NRF2.

There are some limitations to this work. For one, this study was centered around the effects of hyperoside in CML cell lines, but studies of imatinib-resistant CML cells may be of greater clinical relevance and will be an important focus for future research. Hyperoside treatment of mice will also be necessary to confirm the importance of the NRF2/SLC7A11/GPX4 axis in the anti-leukemic effects of hyperoside.

In conclusion, these analyses offer evidence for the ability of hyperoside to exert its anti-leukemic activity in the treatment of CML. These effects appear to be at least partially mediated by ferroptotic induction through the NRF2/SLC7A11/GPX4 pathway. Hyperoside is thus a promising candidate drug for the treatment of CML.

## Electronic supplementary material

Below is the link to the electronic supplementary material.

Supplementary Material 1

Supplementary Material 2

## Data Availability

No datasets were generated or analysed during the current study.
