# Supplementary material for: Hyperoside induces ferroptosis in chronic myeloid leukemia cells by targeting NRF2
Source: Mol Med. 2024 Nov 21;30:224. doi: 10.1186/s10020-024-01002-7 (PMC11583796; doi:10.1186/s10020-024-01002-7)
Supplement: Supplementary file 1 — Supplementary Material 1 [file 10020_2024_1002_MOESM1_ESM.docx]

**Supplementary file**

**Hyperoside induces ferroptosis in chronic myeloid leukemia cells by targeting NRF2**

**Supplementary Table S1.** Sequences for qPCR primers.

| **Gene Name** | **Primer sequence (5’-3’)** | |
| --- | --- | --- |
| SLC7A11 | Forward | TTTGTTGCCCTCTCCTGCTTTG |
|  | Reverse | AGTGTGCTTGCGGACATGAATC |
| GPX4 | Forward | CCGCTGTGGAAGTGGATGAAG |
|  | Reverse | TGTCGATGAGGAACTGTGGAGAG |
| NADPH | Forward | TCATCACCATTGGCAATGAGCG |
|  | Reverse | CACTGTGTTGGCGTACAGGT |

**
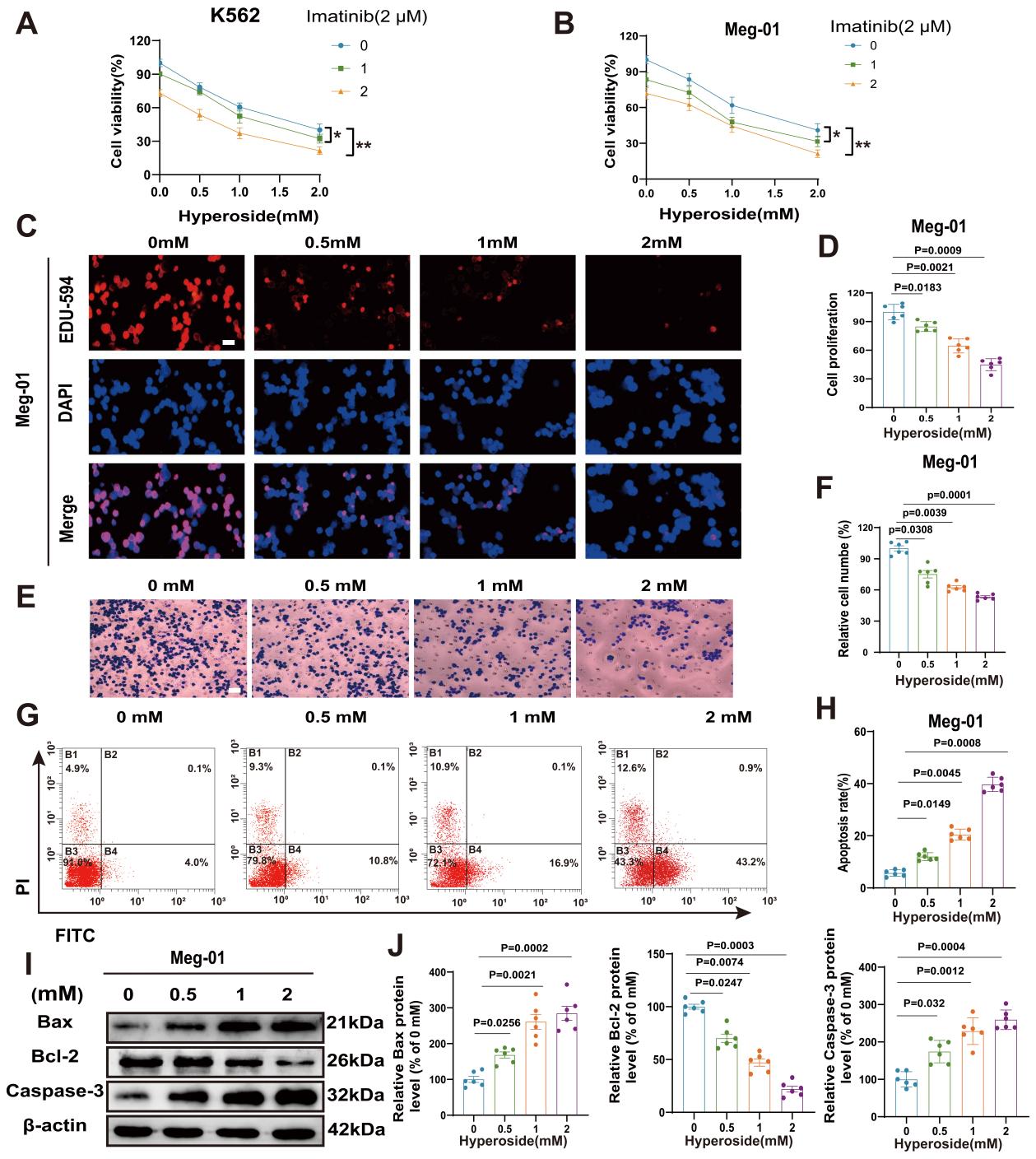
**

**Figure S1.** Hyperoside impairs CML cell viability, migration, and proliferation. **A-B.** K562 and MEG-01 cells were treated with different concentrations of hyperoside and imatinib for 24 h, and cell viability was assayed. **C-D.** Proliferation was detected through an EdU-594 assay. Scale bar: 50 μm. **E-F.** Transwell assays were used to measure migration. Scale bar: 100 μm. **G-H.**  Flow cytometry was used for the quantitative analysis of apoptosis. **I-J.** Bax, Bcl-2, and Caspase-3 protein levels were detected via Western immunoblotting.

**
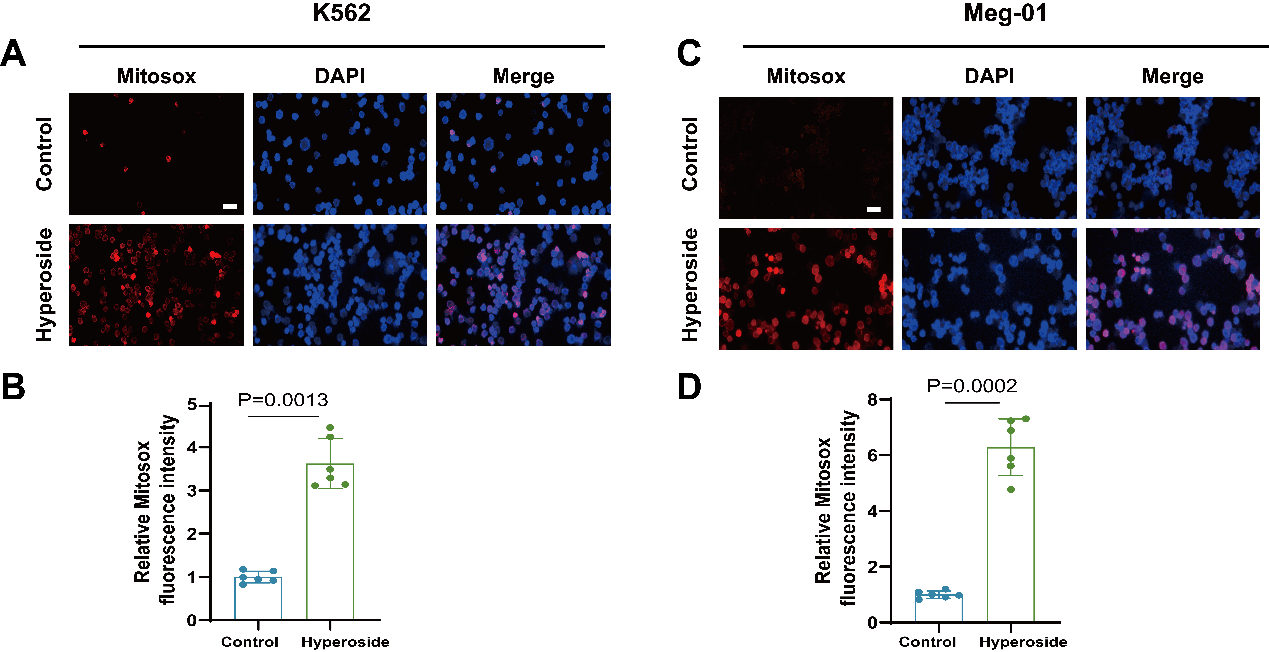
**

**Figure S2.** Hyperoside enhances mitochondrial damage within CML cells. **A-D.** The mitochondrial ROS upon hyperoside treatment were evaluated by MitoSOX staining in K562 and Meg-01 cells.

**
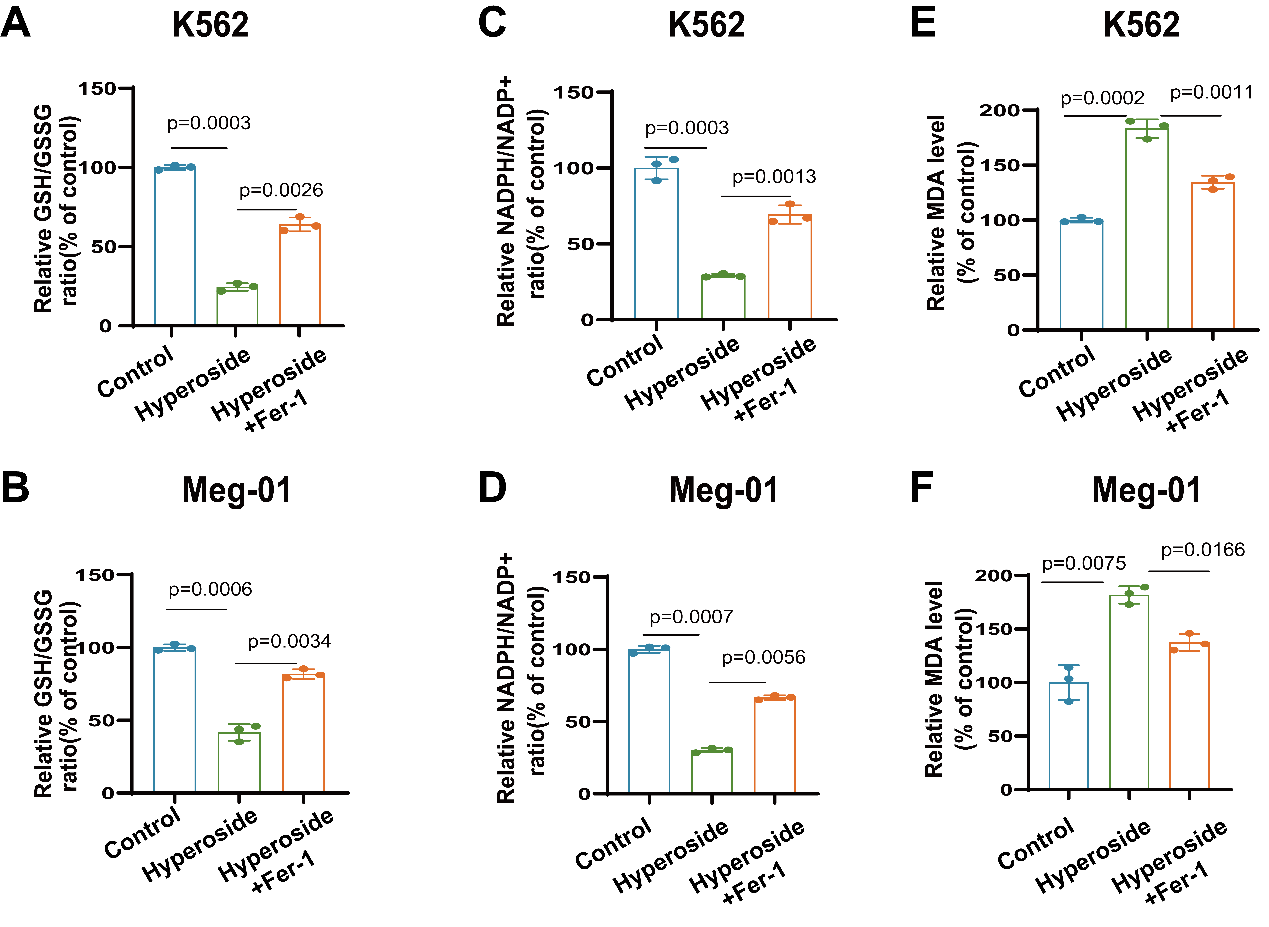
**

**Figure S3.** Ferroptosis is necessary for the anti-leukemic activity of hyperoside in CML. **A-F**.CML cells were incubated with hyperoside (2 mM) with or without Fer-1 (2 μM)，and NADPH/NADP+ ratio, GSH/GSSG ratio, and MDA levels were measured.
