# Supplementary figures and images for: Hyperoside induces ferroptosis in chronic myeloid leukemia cells by targeting NRF2
Source: Mol Med. 2024 Nov 21;30:224. doi: 10.1186/s10020-024-01002-7 (PMC11583796; doi:10.1186/s10020-024-01002-7)

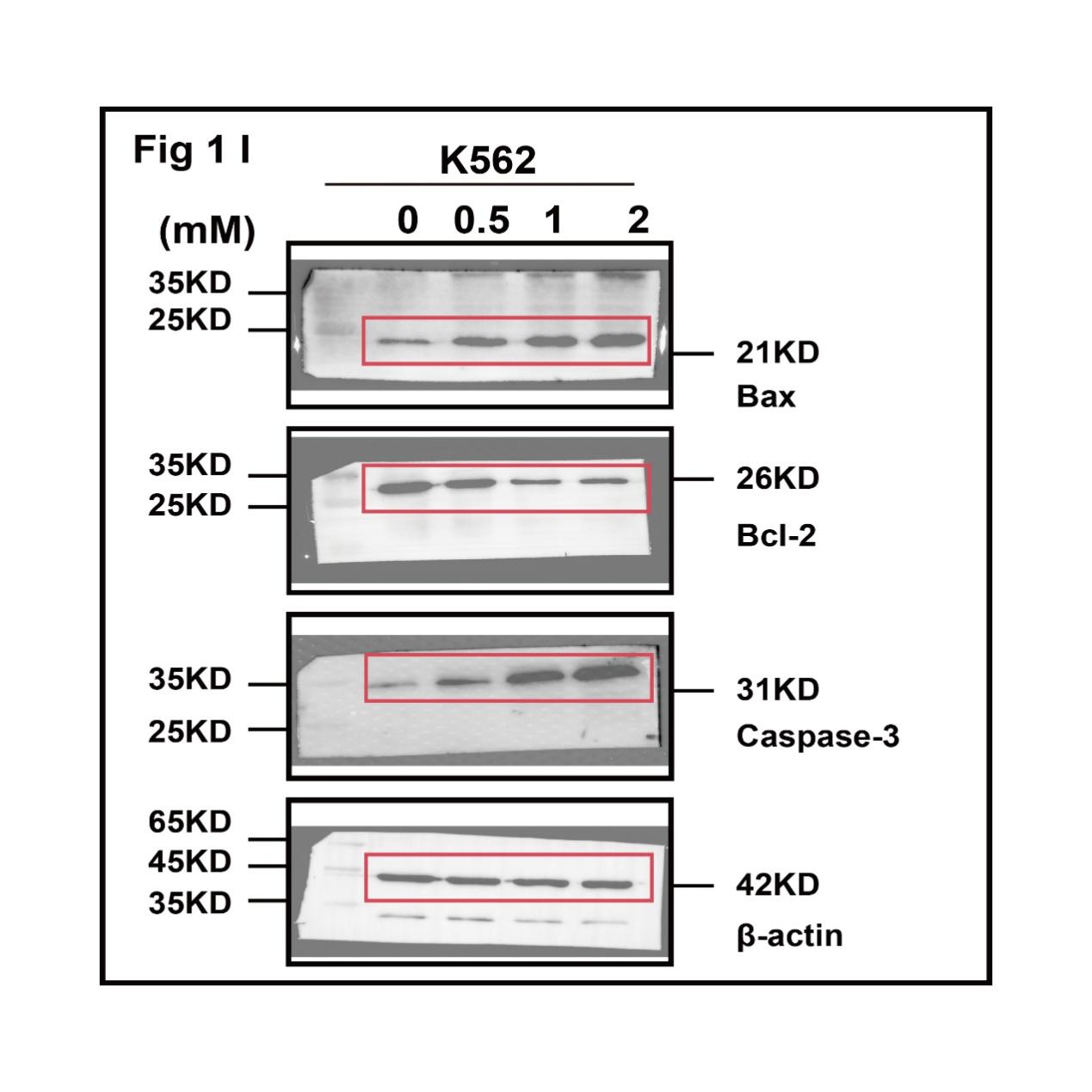

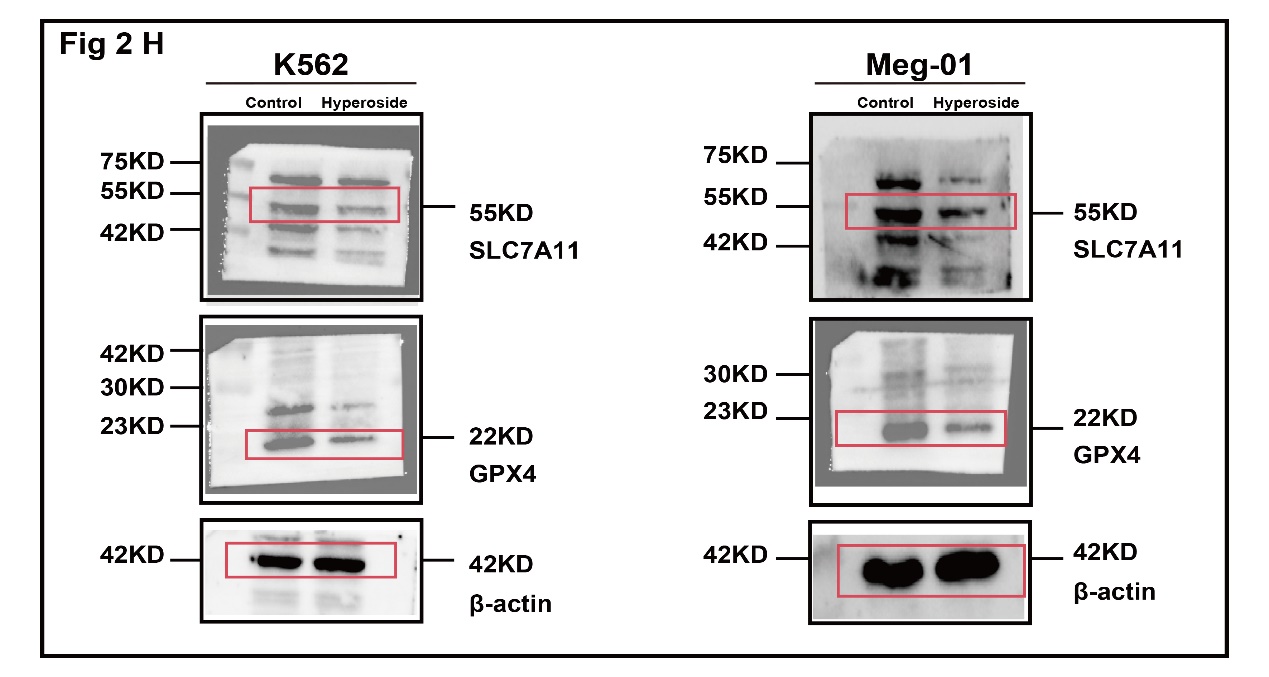

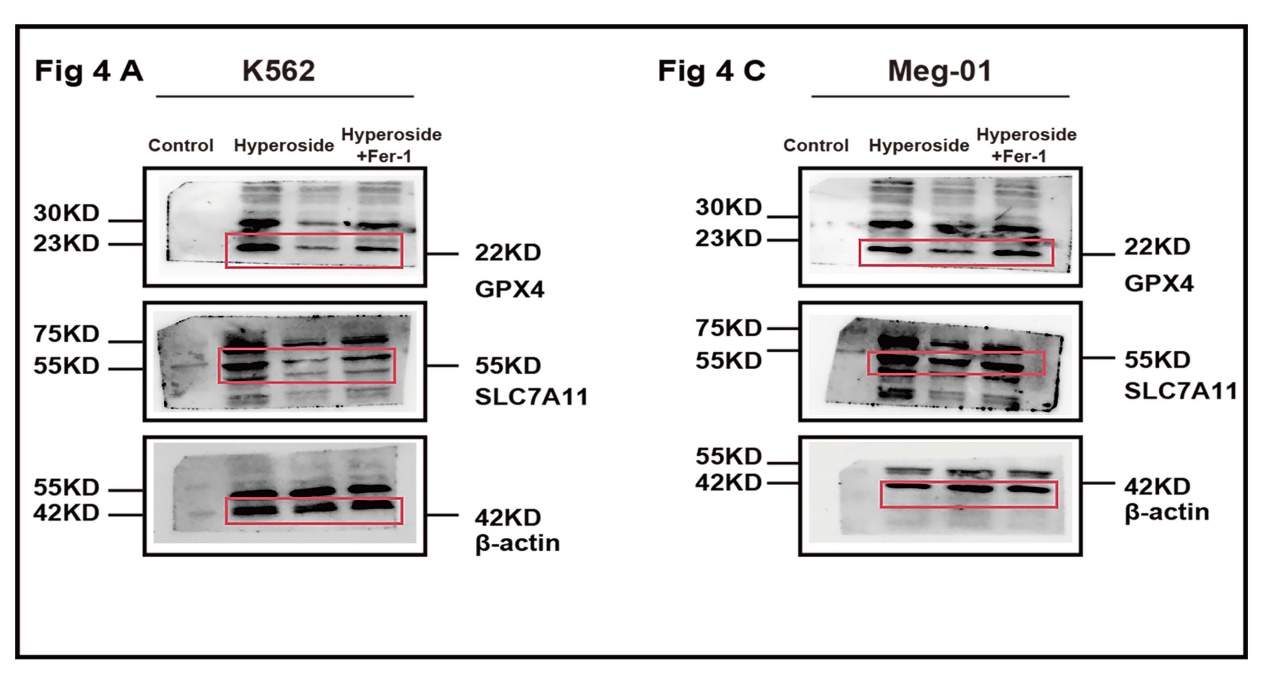

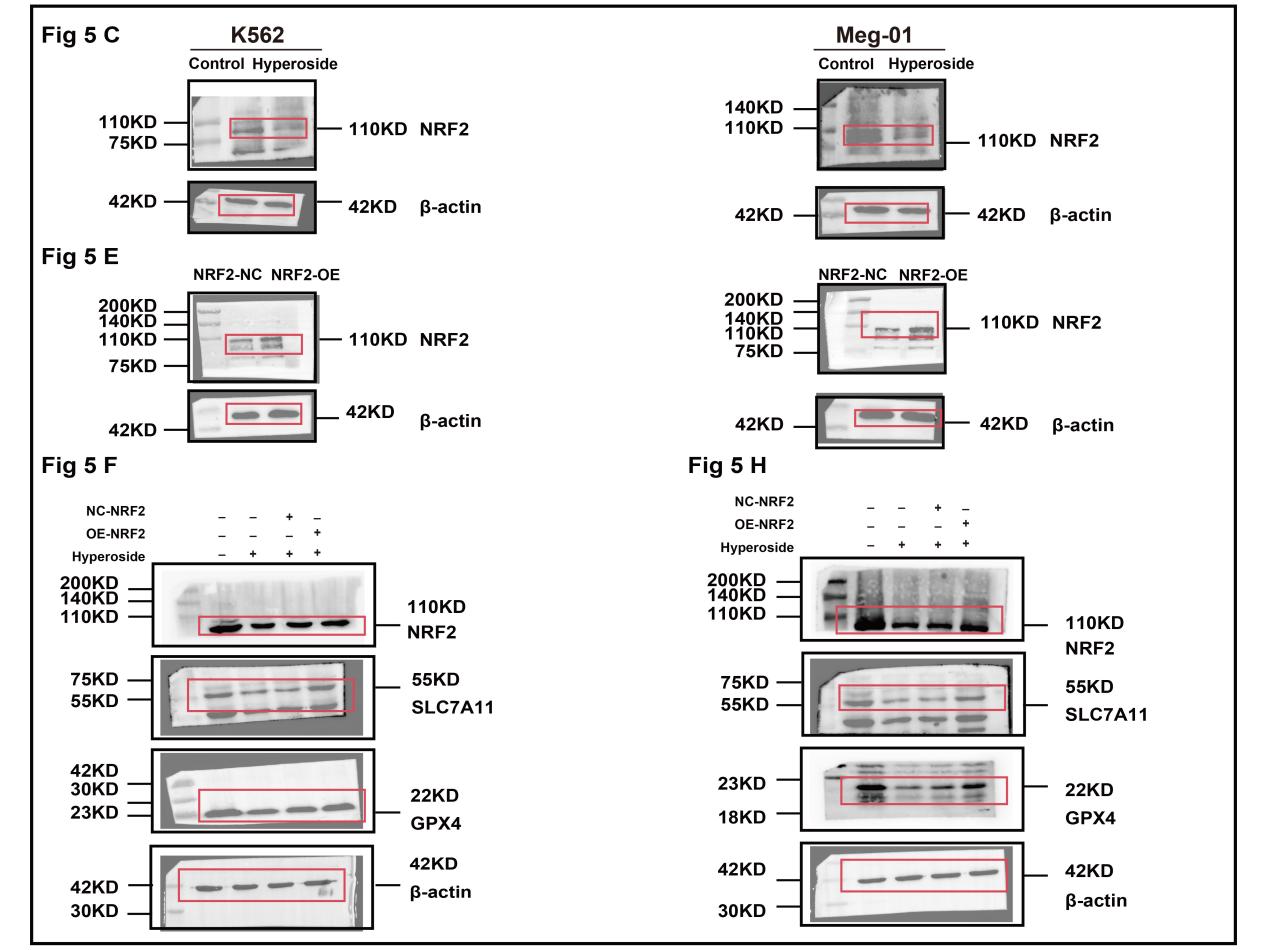

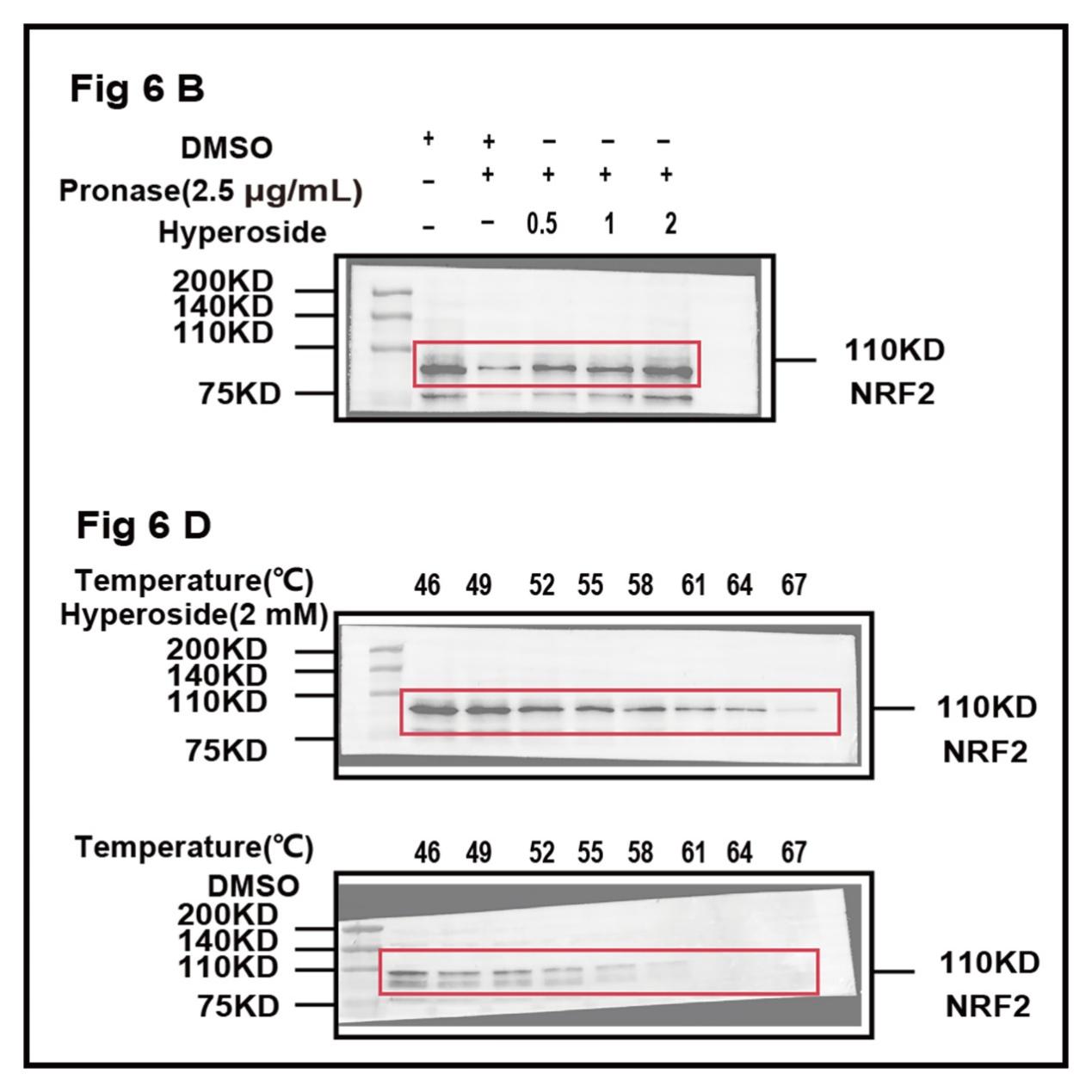

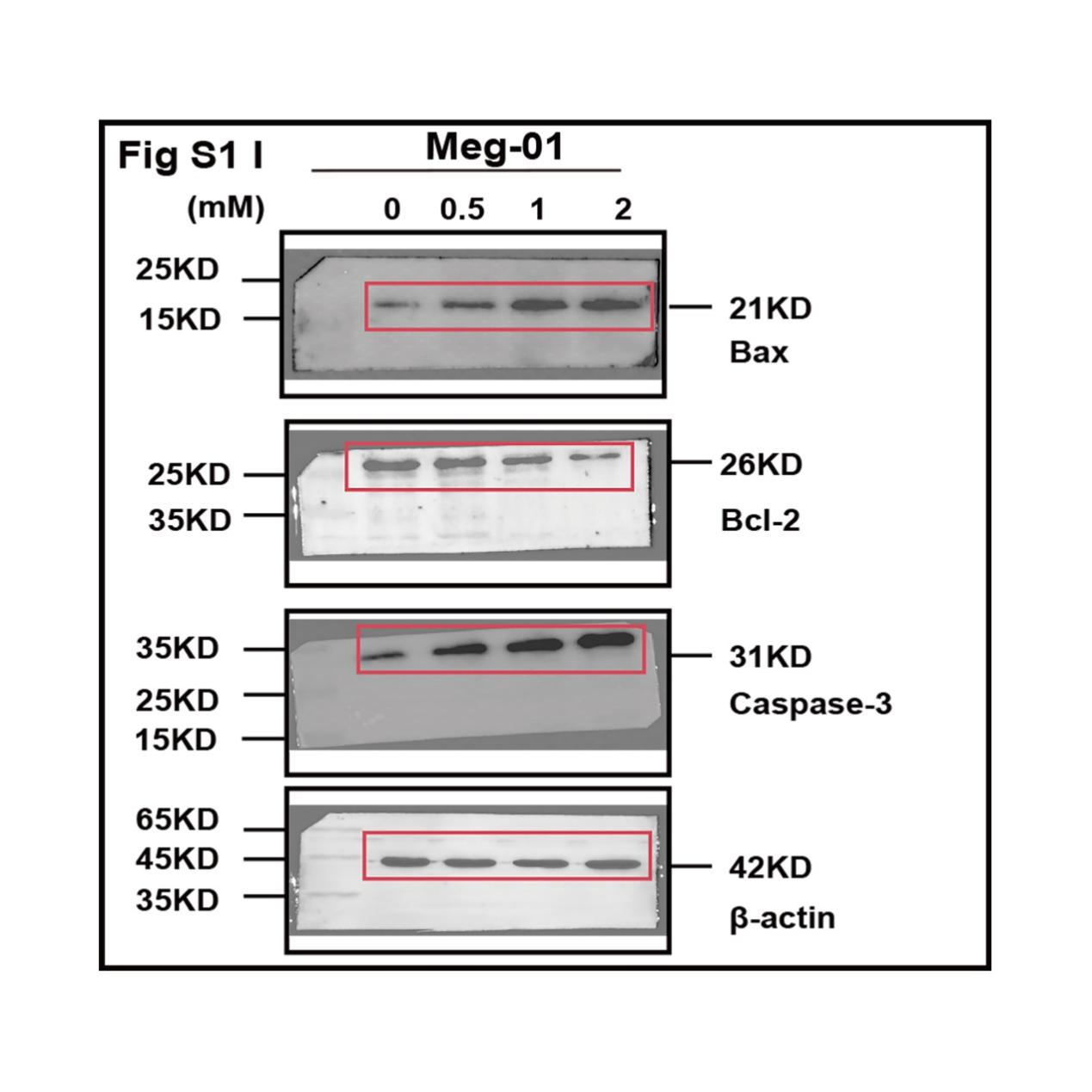

Supplement: Supplementary file 2 — Supplementary Material 2 [file 10020_2024_1002_MOESM2_ESM.docx]
